# Pyorrhœa Alveolaris

**Published:** 1899-07

**Authors:** Burton F. Bishop

**Affiliations:** University of Pennsylvania


### PYORRHŒA ALVEOLARIS.

BY BURTON F. BISHOP, UNIVERSITY OF PENNSYLVANIA.

THE case which I have decided to present is that of a nurse, resident of Philadelphia, Pa., aged thirty-five years. Temperament, nervous. The patient came to the clinic December 9 for the purpose of having her teeth filled; and upon the first examination I discovered that she was wearing an ill-fitting, partial upper, rubber denture, to replace the lateral incisor and two bicuspids on the right side of the arch. On removing the denture I found the gums around the cuspid and two molars very much receded, with a large quantity of salivary calculus deposited on lingual surface of the teeth. By pressing upon the gums pus would flow out at their necks. The two molars were very loose, with their palatine roots nearly exposed to the apical foramen, while the cuspid was rather firm in its socket. A peculiar odor also came from the mouth. On using explorers I found that they could be passed under the gums to nearly the apical foramen. My first treatment consisted of removing the salivary calculus by means of scalers. After thoroughly removing this deposit I injected a three-per-cent. solution of hydrogen dioxide to remove the pus. This was washed out with warm water, and a twenty-five-per-cent. solution of sulphuric acid added to produce healthy granulation. The sulphuric acid, after remaining for a minute or two, was neutralized by bicarbonate of soda, and washed out with warm water.

Sulphate of quinine was then packed into the pockets by means of an orange-wood stick sharpened to a thin point. I then instructed the patient to use an antiseptic mouth-wash, which consisted of

℞ Hydronaphthol, gr. xv;
Alcoholis,
Aquæ destillatæ, āā ℥i. M.

Sig.—Twenty to thirty drops to a glass of water, to be used three times a day.

On her second return I found the pus-pockets much smaller, teeth firmer in their sockets, and less pus flowing out on pressure. I gave her the treatment described above, with the exception of sulphuric acid, as this would have destroyed the healthy granulation which had already taken place.

The patient returned six times, and at each visit a change for the better was noticeable. At last visit no pus was present, with the gums practically in contact with the teeth.

This disease has been known in the family for three generations.

